# Peritoneal tumor spread in serous ovarian cancer-epithelial mesenchymal status and outcome

**DOI:** 10.18632/oncotarget.3746

**Published:** 2015-05-11

**Authors:** Katharina Auer, Anna Bachmayr-Heyda, Stefanie Aust, Nyamdelger Sukhbaatar, Agnes Teresa Reiner, Christoph Grimm, Reinhard Horvat, Robert Zeillinger, Dietmar Pils

**Affiliations:** ^1^ Department of Obstetrics and Gynecology, Comprehensive Cancer Center (CCC), Medical University of Vienna and Ludwig Boltzmann Cluster Translational Oncology, Vienna, Austria; ^2^ Department of Pathology, Medical University of Vienna, Vienna, Austria

**Keywords:** epithelial ovarian cancer, peritoneal tumor spread, flow cytometric analysis, RNA-sequencing, next generation sequencing

## Abstract

In this study we aimed to analyze the biological mechanisms underlying apparently different modes of peritoneal tumor spread in serous ovarian cancer: miliary (widespread, millet-like lesions) versus non-miliary (bigger, exophytically growing implants). Tumor tissues and ascites from 23 chemotherapy naive patients were analyzed by RNA-sequencing and flow cytometry. On the basis of differential gene expression between miliary and non-miliary, gene signatures were developed. A calculated tumor spread factor revealed a significant independent negative impact of miliary spread on overall survival (HR 3.77; CI_95_ 1.14–12.39; *p* = 0.029) in an independent cohort of 165 serous ovarian cancer patients. Comparing previously published epithelial-mesenchymal transition (EMT) gene signatures, non-miliary spread correlated significantly with a reduced epithelial status. We conclude that serous ovarian cancer is a heterogeneous disease with distinct modes of peritoneal tumor spread, differing not only in clinical appearance, but also in molecular characteristics and outcome.. EMT, peritoneal inflammation status, and therapeutic options are discussed.

**Significance:**

More than half of serous epithelial ovarian cancer patients present with a newly described type of intraperitoneal tumor spread, associated with differences in the inflammation status, activated oncogenic pathways, lack of EMT, and thus reduced overall survival. Both, the diminished immune reaction and the enhanced epithelial and malignant characteristics of the tumor cells open new avenues for therapeutic options and strategies, like Catumaxomab, already in clinical use.

## INTRODUCTION

Epithelial ovarian cancer (EOC) is the sixth leading cause of death among women with malignancies worldwide and the leading cause of death from gynecological cancers [1]. Median survival time over all stages is only 4.5 years due to the advanced stage at first diagnosis caused by an initially asymptomatic course of the disease. Current standard treatment strategies involve primary cytoreductive surgery followed by a platinum and taxane based chemotherapy [2], increasingly supplemented with angiogenesis inhibiting drugs like bevacizumab [3]. Nevertheless, no specific treatments according to subclassification approaches are currently in use.

Similar to molecular subclassification approaches in breast cancer [4, 5], several attempts to classify EOC according to molecular characteristics were made over the past years [6–9] but with limited impact on clinical routine. We validated one such molecular subclassification by Yoshihara *et al*. in a cohort of 165 FIGO II/III/IV serous EOC patients. Besides this molecular subclassification, peritoneal carcinomatosis was shown to be the most important predictor for overall survival [10].

In contrast to other cancer entities, EOC spreads predominantly in the peritoneal cavity, often accompanied by massive production of (malignant) ascites. Although small amounts of peritoneal fluid are present in healthy women as well, increasing volumes of ascites might generate a favorable tumor microenvironment, enabling the characteristic patterns of transcoelomic tumor spread in ovarian cancer [11]. Studies have shown that despite frequent involvement of the local lymph system, extra-peritoneal (so-called distant) metastases are rare and occur predominantly late [12].

In order to metastasize, ascitic tumor cells have to evade programmed cell death following cell detachment (anoikis). Both, cell aggregation and epithelial-mesenchymal transition (EMT) are strategies to overcome anoikis [13]. EMT was described as key event in cancer progression and metastasis. This may not be a binary but rather a dynamic, continuous process as intermediate states of cells expressing both, epithelial and mesenchymal markers, were described [14]. Recently, an EMT spectrum with corresponding gene signatures in various cancer entities was introduced and used to assess chemotherapy resistance and overall survival [15, 16].

In this study, we focused on two macroscopically different types of tumor spread which can be distinguished during surgery: i) one exhibiting millet sized lesions looking very similar to tuberculosis peritonitis [17] referred to as *miliary* tumor spread, ii) the other characterized by few, much bigger and exophytically growing implants (*non-miliary*). This motivated us to assess cell surface marker characteristics and whole genome transcriptomes in high grade serous ovarian cancer (HGSOC) patients and use bioinformatic analyses to identify the most prominent involved pathways. Gene signatures for miliary and non-miliary tumor spread were developed and used to determine the impact of the tumor spread behavior on patients' outcome.

## RESULTS

**Figure 1:**
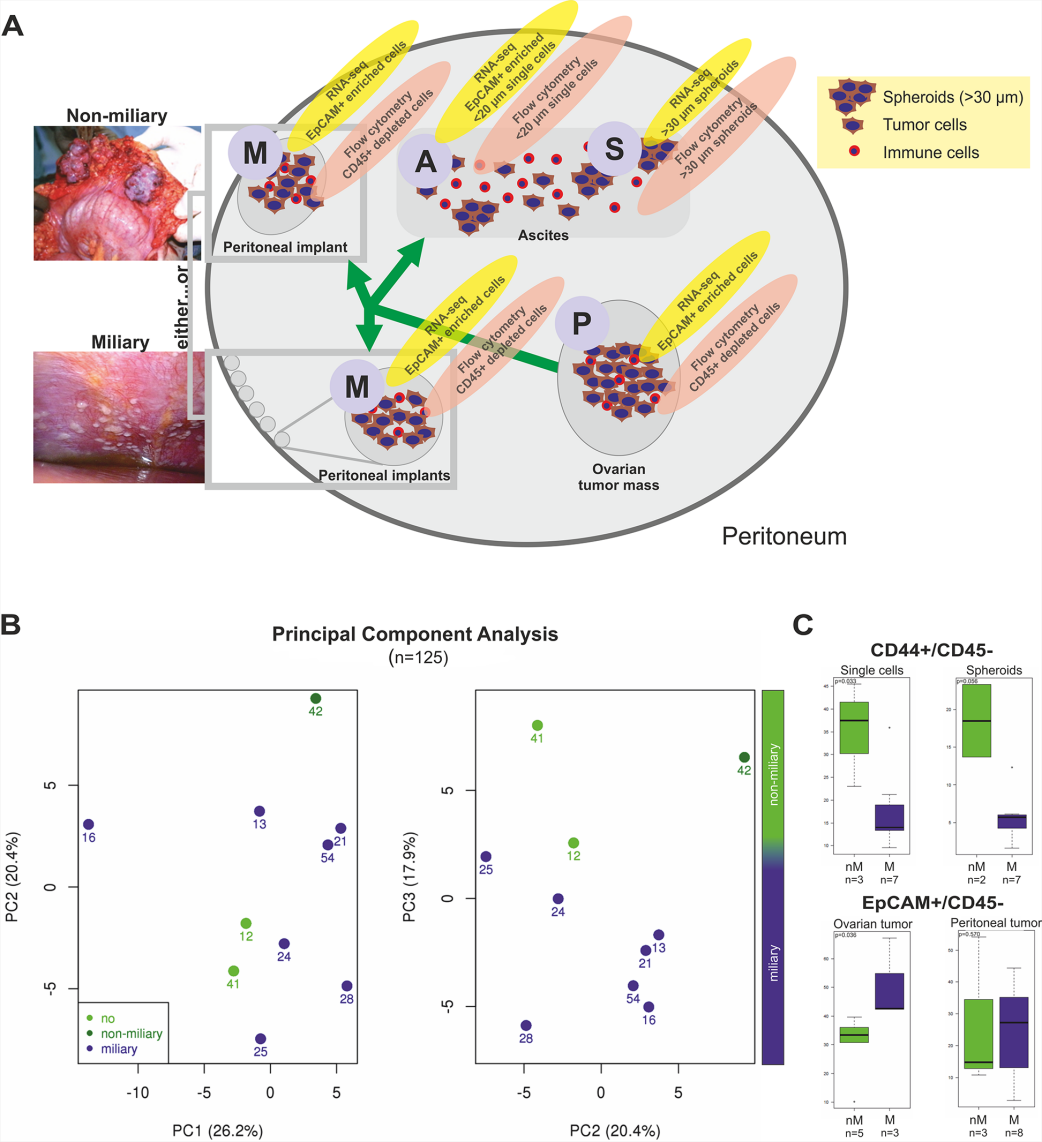
**A. Scheme, showing all types of tissues and methods used in this study.** Ovarian tumor mass (P, primary tumor), peritoneal, tumor mass (M, metastasis) of both tumor spread types, miliary and non-miliary, ascites single cells (A) and ascites spheroids (S) were collected. Solid tumors were disaggregated. Ascites cells were separated by filtration into ascites single cells and spheroids, i.e. tumor cell aggregates. For RNA-sequencing, P, M, and A samples were enriched for EpCAM+ cells, while S samples were not further enriched. For flow cytometry, cells from disaggregated P and M tissues were depleted of CD45+ cells, S samples were disaggregated, and A samples were analyzed without further treatment. **B.** Principal Component Analysis (PCA) of all flow cytometric determined subpopulation frequencies (CD133+, CD44+, EpCAM+, and L1CAM+; *n* = 125; cf. Supplementary Tables S2) from ascites single cells, ascites spheroids, ovarian tumor masses, and peritoneal tumor masses. Each dot represents one patient. Light green indicates patients without peritoneal metastases, green, patients of the non-miliary tumor spread type, and blue, patients of the miliary tumor spread type. For statistical feasibility, only patients with available ascites samples were included. **C.** Boxplots showing the frequencies (in percent with reference to live cell counts) of CD44+/CD45− cells in ascites single cell and spheroid samples (upper graph) and of EpCAM+/CD45− cells in ovarian and peritoneal tumor masses (lower graph). In green, samples of non-miliary tumor spread (nM) and in blue samples of miliary tumor spread (M) are shown. *P*-values were calculated with Wilcoxon rank-sum tests. CD45+ cells, dead cells, and cell doublets and triplets were excluded from further analysis.

In clinical routine peritoneal carcinomatosis is defined as widespread metastasis of cancerous tumors on the surfaces of the abdomen [18]. Some FIGO III patients do not have detectable peritoneal implants, but retroperitoneal lymph node metastases (which render them FIGO IIIC or FIGO IIIA/1 using FIGO 2014 [19]). The definition for miliary and non-miliary is shown in Table 1 and exemplified in Figure 1A. Clinical and pathological characteristics of 23 HGSOC patients enrolled in this study are shown in Supplementary Table S1. All except for one carry functional *TP53* mutations. Analyzed tissues (ascites single cells (A), ascites aggregated tumor cells/spheroids (S), solid ovarian (primary) tumors (P), and solid peritoneal (metastatic) tumors (M)) and methods are outlined in Figure 1A.

**Table 1:** Definition of the proposed classification of peritoneal tumor spread behavior

Spread type	Peritoneum	Size	Lymph Nodes^1^	Code
Miliary (M)	> 20 implants	most of them < 2 cm	pNX, pN0, or pN1	2
Non-miliary (nM)	no implants/few implants	> 2 cm	pN1/pNX, pN0, or pN1	0/1
Indeterminable (due to late stage)	widespread, high tumor load	>> 2 cm or adnate	pNX, pN0, or pN1	

1 pN0 = no regional lymph node metastasis, pN1 = regional lymph node metastasis, pNX = regional lymph nodes not assessed

### Flow cytometric surface marker evaluation

Flow cytometric analysis was performed with a total of 38 samples from ascites (A/S) and solid tumor tissues (P/M) from 15 patients. Frequencies for 125 subpopulations of cells positive for combinations of surface proteins CD45, EpCAM, CD133, L1CAM, and CD44 were determined (Supplementary Table S2). The gating strategy is outlined in Supplementary Figure 1 and all subpopulation frequencies are given in the Table “FC_PCA_loadings.xlsx” in the Supplementary data zip-file. Correlation of these subpopulations with the mode of peritoneal tumor spread revealed an over-representation of CD44+/CD45− cells in non-miliary in both, A and S (median 37.5% versus 14.0%, *p* = 0.033 and 18.5% versus 5.7%, *p* = 0.056, respectively; Figure 1C). Furthermore, the EpCAM+/CD45− population was higher in miliary compared to non-miliary P, but not in M samples (median 42.6% versus 33.4%, *p* = 0.036; Figure 1C). Using all 125 cell-type frequencies (including only patients with available ascites samples), miliary could be separated from non-miliary in a principal component analysis (PCA) along PC3 (Figure 1B). To show cell types mainly responsible for this component, the loadings for PC3 revealed CD44+/CD45− in A/S samples and interestingly the CD133+ population in M samples (more abundant in miliary, median 1.96% versus 0.15%, *p* = 0.028) as the most important cell types (see “FC_PCA_loadings.xlsx”).

Given the differences in cell composition between miliary and non-miliary tumor tissues and taking into account the usually heterogeneous cell composition of tumor tissues we decided to enrich for EpCAM positive cells prior to transcriptome analysis to avoid biasing effects from stromal and other microenvironmental cells.

### Transcriptome analysis with RNA-sequencing and functional annotation

Total RNA from 42 tumor cell enriched tissue samples (P, M, A, and S) from 21 patients were sequenced to a median depth of 26.03 million 50 bp paired-end reads (range: 10.67 - 44.65 million). After filtering of putative reads from circular RNAs [20] reads were mapped and counted into a gene model. Finally, 28 203 reliably expressed coding and non-coding genes were used for further analyses (see Supplementary methods).

To reduce the impact of the tumor microenvironment like stromal or infiltrating immune cells, solid tissues were enzymatically digested and enriched for EpCAM positivity to ensure the comparability of tumor cells from ascites and solid tumors. The same enrichment was performed with single ascites cells, whereas tumor cell aggregates were isolated solely by filtration. EpCAM staining of agarose and paraffin embedded ascites tumor cells showed that cell aggregates in malignant ascites are mainly composed of tumor cells with only very few infiltrating or associated immune cells, thus comparable to EpCAM enriched single tumor cells.

### Modes of tumor spread: miliary versus non-miliary

**Figure 2:**
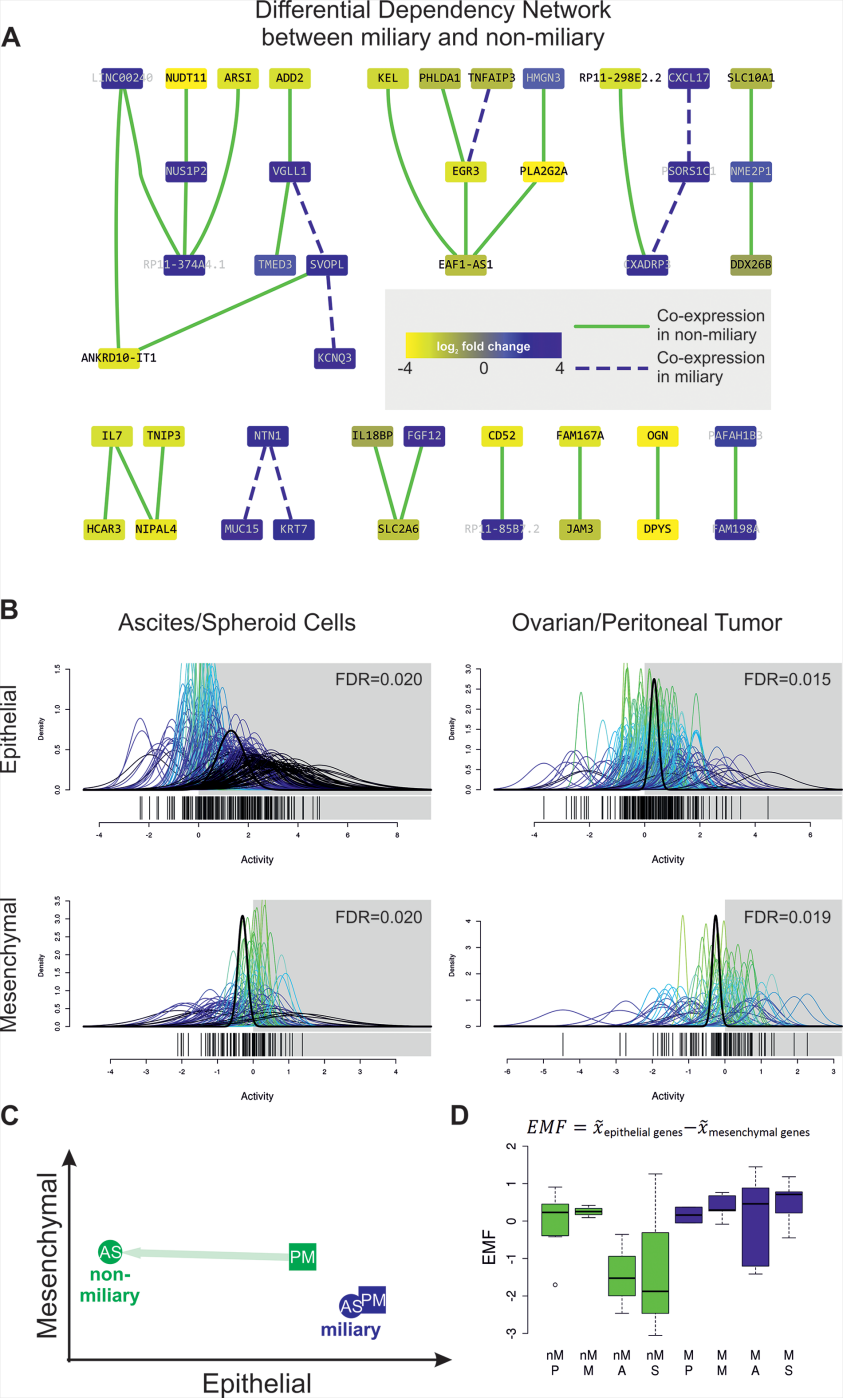
**A. Differential Dependency Network (DDN) between miliary and non-miliary samples constructed from the 90 significantly differentially expressed genes between both tumor spread types.** Colors of nodes represent log_2_ fold changes between both conditions (blue, up in miliary). Green solid and blue dashed edges indicate significantly different connections, defined by co-expression in samples of the respective type, miliary and non-miliary. **B.** Quantitative set analysis using an epithelial and a mesenchymal gene signature [16] and ascites single tumor cells or spheroids (A/S, left) and ovarian or peritoneal solid tumor masses (P/M, right) from patients with miliary tumor spread compared to patients with non-miliary tumor spread (QuSAGE). The x-axis (“Activity”) represents probability density functions (pdf) for every fold-change between the corresponding comparison of every gene in the gene-set (colored) and a combined probability density function over all genes in the gene set (bold black). A pdf around 0 indicates no change in the activity of the gene set in the corresponding comparison. **C.** Scheme depicting epithelial and mesenchymal characteristics of tumor cells, according to the calculated EMF. **D.** Boxplot showing the epithelial-mesenchymal factor for non-miliary (nM, green) and miliary (M, blue) samples of the different tissue origins. EMF is calculated as median expression ($\overset{¯}{\text{x}}$) of epithelial genes minus median expression ($\overset{¯}{\text{x}}$) of mesenchymal genes and is interpreted as relative differences between samples.

No genes were significantly differentially expressed between single ascites cells (A, *n* = 12) and tumor cell aggregates (S, *n* = 10) or solid tumors from the ovary (P, *n* = 10) and implants from the peritoneum (M, *n* = 10), combining the samples from the same and different patients. Therefore, all A and S and also all P and M samples were analyzed together. In contrast, 6 519 genes were significantly differentially expressed between all AS (*n* = 22) and PM (*n* = 20) samples. In an independent analysis, comparing gene expression between miliary samples (*n* = 25) and non-miliary samples (*n* = 17) and taking all tissue origins into account (in the design matrix for significance analysis, see Supplementary methods), 90 genes were found to be significantly differentially expressed (FDR 10%), 49 down- and 41 upregulated in miliary (Table 2, “SignificantDiff_Genes.xlsx”). The majority of these genes (72%) were protein coding. A first interpretive look at the difference between miliary and non-miliary by constructing the Differential Dependency Network (DDN) from these 90 differentially expressed genes revealed axes of known oncogenes, *KRT7-NTN1-MUC15* [21–23], *CXADRP3-PSORS1C1-CXCL17* [24], and *VGLL1-SVOPL-KCNQ3* [25], upregulated in miliary and an axis of genes of mostly unknown cancer-relevance, *HCAR3-IL7-NIPAL4-TNIP3*, upregulated in non-miliary (Figure 2A). Interestingly, IL7 is a mediator of chronic (humoral and cellular) inflammation [26].

**Table 2:** Numbers of (A) significantly up- and downregulated genes and gene sets and (B) significantly activated and inhibited pathways

A)				Significant genes		Significant gene sets^1^ (qusage)						
Test	Sample subset	FDR	Direction	Protein coding	Non-coding	C1	C2	C3	C4	C5	C6	C7
Miliary vs non-miliary	All	10%	Up	33	8	43	269	7	89	104	2	19
			Down	32	17	101	2 150	404	315	699	117	1 046
Miliary vs non-miliary	A,S	10%	Up	59	18	1	3	0	1	2	1	2
			Down	110	28	0	12	0	1	5	0	1
Miliary vs non-miliary	P,M	10%	Up	2	0	67	499	20	131	164	5	37
			Down	0	0	106	2 451	365	425	813	114	1 370
AS vs PM	Non-miliary	5%	Up	641	222	3	389	11	80	63	1	423
			Down	510	164	5	246	49	24	63	4	19
AS vs PM	Miliary	5%	Up	94	28	1	132	8	52	23	0	146
			Down	296	179	4	267	84	11	34	6	39
**From total:**				**16 674**	**11 529**	**326**	**4 722**	**836**	**858**	**1 454**	**189**	**1 910**

B)		SPIA				PAGI	
Test	Sample subset	FDR	Direction	Pathways (Supp. Figure)	Number of used genes	FDR	Pathways
Miliary vs non-miliary	All	10%	Activated	0	845	n.d.	
			Inhibited	3 (S6)	(FDR 20%)		
Miliary vs non-miliary	A,S	10%	Activated	5 (S7)	215	1%	76
			Inhibited	6 (S7)			
Miliary vs non-miliary	P,M		Activated	n.d.^2^	(2)	1%	11
			Inhibited	n.d.			
AS vs PM	Non-miliary	5%	Activated	16 (S8)	1 537	n.d.	
			Inhibited	7 (S8)			
AS vs PM	Miliary	5%	Activated	8 (S9)	597	n.d.	
			Inhibited	0			
		**From total:**	**156**			**219**	

1 C1, positional gene sets; C2, curated gene sets; C3, motif gene sets; C4, computational gene sets; C5, GO gene sets; C6, oncogenic signatures; C7, immunologic signatures.

2 n.d., not determined.

A non-linear dimensionality reduction approach (Isomap) using only the 30 most significantly differentially expressed genes (FDR 5%) between miliary and non-miliary samples revealed a separation of P/M- and A/S-samples along dimension two. Furthermore, miliary and non-miliary could be discriminated using dimensions 1 and 2 (Supplementary Figure S2). This means, genes which are important for tumor spread also differ between solid (P/M) and floating (A/S) tumor tissues.

As opposed to solid tumor tissues (P/M) with only two significantly upregulated genes (*ACKR2, CYP8B1*) between miliary and non-miliary (9 versus 11 samples, respectively), tumor cells from ascites (A/S, 16 miliary versus 6 non-miliary samples, respectively) revealed 215 significantly differentially expressed genes, 138 of them down- and 77 upregulated in miliary (Table 2). Among the most downregulated genes were matrix metalloproteinases *MMP12* and *MMP9* with log_2_ fold-changes of −4.8 and −4.6, respectively. In contrast, significant differences in gene set expressions were tremendously higher in P/M samples (6 567 gene sets) compared to A/S samples (29 gene sets), most of them down-regulated in miliary (85.9%). This surprising result from subtle downregulation of many genes, instead of strong and significant deregulation of only a few genes could be indicative for a global modification of cell characteristics. Interestingly, “Oxidative phosphorylation in mitochondria” (C2) and “response to oxidative stress” (C5) were among the most significantly upregulated gene sets and the Reactome pathway “glucose metabolism” (C2) among the most significantly downregulated gene sets in miliary.

Pathway analysis of the differentially expressed genes in ascites using SPIA analysis yielded eleven deregulated pathways (FDR 10%), many of them including *NF-κB* and *PI3K* and some with *MMP9* and *IL8* as deregulated key players (“SPIA.xlsx” and Folder “AS_nM_M” in the Supplementary zip-file). “Pathways in cancer“, “Proteoglycans in cancer”, and signaling pathways “NOD-like receptor signaling”, “TNF signaling”, and “Ras signaling” were activated in miliary, whereas “Cytokine-cytokine receptor interaction”, “Chemokine signaling pathway”, the signaling pathways “ErbB signaling” and “Estrogen signaling”, together with “Mineral absorption”, and “Epithelial cell signaling in *H. pylori* infection” were inhibited (see Folder “pathways” in Folder “AS_nM_M”). This indicates that some oncogenic pathways like the TNF- and the RAS-pathway are activated and others like the ErbB- and the estrogen-pathway are inhibited in miliary. Inhibition of the ErbB and the estrogen pathway as well as cytokine-cytokine interactions and chemokine signaling indicates that ascites tumor cells in miliary became more independent from typical growth (inhibition) signals. In solid tumor cells a more sensitive method for identifying deregulated KEGG pathways by including information about the crosstalk *between* pathways (PAGI) revealed eleven deregulated pathways between the two spread types: “Pancreatic secretion”, “Carbohydrate digestion and absorption”, “Glycolysis and Gluconeogenesis”, “Fructose and mannose metabolism”, “Starch and sucrose metabolism”, “Retinol metabolism”, “Metabolism of xenobiotics by cytochrome P450”, “Drug metabolism - cytochrome P450” (all involved in cell metabolism), and “Cell cycle”, “Oocyte meiosis”, and “Steroid hormone biosynthesis” (Table “SPIA.xlsx”). In addition, the PAGI package evaluated the global influence factor (GIF) score in the global gene-gene network constructed based on the relationships of genes extracted from each pathway in the KEGG database and the overlapped genes between pathways. In Supplementary Figure S4 the 30 genes with the highest GIF values in each comparison are shown (upper figure) together with boxplots over tissues and spread types of gene expression values from the three genes with the highest GIF (i.e. highest influence on the KEGG pathways-network) in each of the comparisons or in both (lower figure).

**Figure 3:**
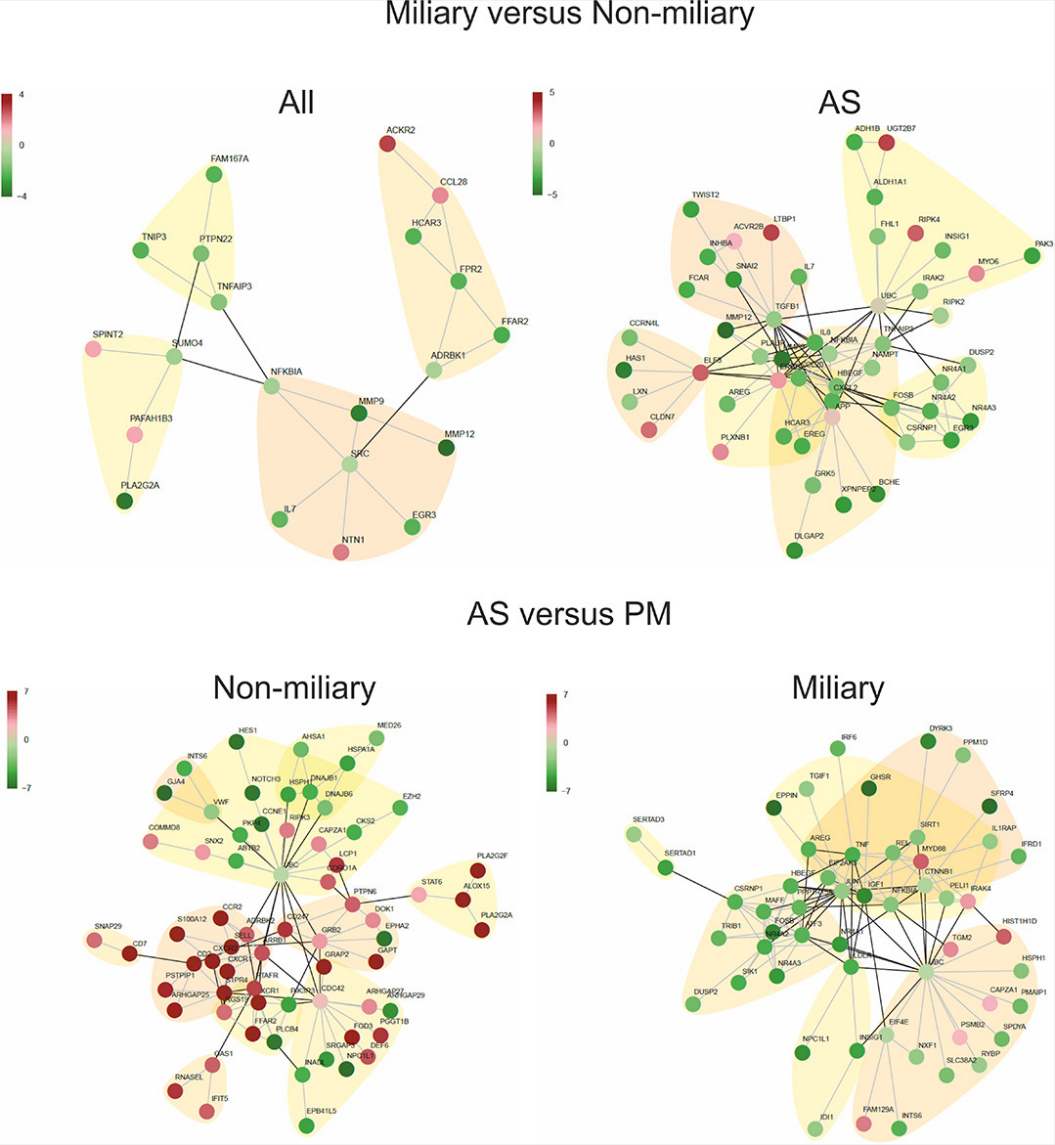
High scoring protein interaction networks Based on differentially expressed genes between miliary and non-miliary spread in all analyzed tissue samples and in ascites samples only (A/S) (upper panel) and high scoring networks based on differentially expressed genes between solid tumors (P/M) and ascites tumor cells (A/S) in non-miliary and miliary samples, separately (lower panel). Edges represent published evidence of interactions between two proteins (STRING 9.1 database). Red, upregulated and green, downregulated in tumors from patients with miliary tumor spread.

Network analysis using STRING v9.1, comprised of functional protein interactions, uncovered a high-scoring protein network in ascites relevant for intraperitoneal tumor spread, consisting of genes involved in EMT, epithelial cell characteristics, and steroid-thyroid hormone-retinoid receptor activity, as well as *NFKBIA*, *IL8*, *TGFB1*, *ERBB2*, *MMP9*, and *MMP12* as hub-genes (Figure 3). Focusing only on experimentally validated protein-protein interactions (PPIs), the high-scoring network includes *C9orf169, ZNF587, TRAF1* (TNF receptor-associated factor 1 which together with TRAF2 is required for TNF-alpha-mediated activation of MAPK8/JNK and NF-κB), and *REL* (encodes c-Rel, a transcription factor that is a member of the Rel/NFKB family, thus a paralog of NFKB1) as important hub-genes (Supplementary Figure S5). A similar STRING analysis comparing all miliary samples with all non-miliary samples (A/S/P/M) revealed mainly *NFKB1A*, *SRC* and *SUMO4* (Small ubiquitin-related modifier 4, conjugates to IKBA and negatively regulates NF-κB transcriptional activity) as relevant hub-genes (Figure 3). Using only experimentally verified PPIs, *LNX1* (Ligand of Numb-Protein X, an E3 ubiquitin-protein ligase) was discovered as hub-gene and a sub-network of six keratins was shown to be upregulated in all miliary tumor cell types (Supplementary Figure S5).

Gene signatures for miliary and non-miliary were defined using transcriptional differences between the two tumor spread types of P/M tumor tissues, *n* = 9 and *n* = 11, respectively, using a method proposed by Abbas *et al*. [27], which is independent from significantly differentially expressed genes: one consisting of 110 genes upregulated in miliary and one consisting of 162 genes upregulated in non-miliary (“Spread_gene_signatures.xlsx”). The reasoning for using only P/M tissues was that these gene signatures could subsequently be used for validating the impact of the spread-characteristics on patients' outcome using microarray whole genome expression data from solid tumor tissues (see below). Using these gene sets a simple but robust “Tumor Spread Factor“ (TSF) was calculated: Median expression of the miliary genes minus median expression of the non-miliary genes, representing the preference for miliary (if high) or non-miliary (if low) (Supplementary Figure S6C).

Among the miliary gene set, 64 of 110 genes were coding (58.2%) and 46 non-coding and among the non-miliary gene set, 95 of 162 genes were coding (58.6%) and 67 non-coding. The coding genes of both sets were annotated with disease ontology (DO) terms and a network was built using these annotations. Both gene sets are enriched for cancer associated terms and “disease of cellular proliferation”. Additionally, the non-miliary gene set is enriched with the terms “immune system” and “reproductive system disease”. In Supplementary Figure S6A these annotation networks are shown.

### Epithelial and mesenchymal characteristics of tumor cells

As prominent EMT genes were detected among spread type associated genes, published epithelial (212 genes) and mesenchymal (96 genes) gene signatures [16] were used for characterization of tumor cells. A simple and robust ”Epithelial-Mesenchymal Factor” (EMF) similar to the TSF (see above) was calculated (Figures 2B,2C,2D).

A Quantitative Set Analysis for Gene Expression (QuSAGE) using the epithelial and mesenchymal gene sets revealed that both, A/S as well as P/M samples, were significantly more epithelial-like in miliary (*n* = 16 and *n* = 9, respectively) and significantly more mesenchymal-like in non-miliary (*n* = 6 and *n* = 11, respectively; Figure 2B and Table “qusage.xlsx”). Albeit the differences are significant, they are not very strong indicating that peritoneal tumor spread (represented by the TSF) and the EMF are somewhat correlated (*r* = 0.28, *p* < 0.001) but not identical factors. Only six genes (2.2%) overlap between the genes of both factors. Consistently, in non-miliary A/S samples the EMF is lower (i.e. less epithelial-like or more mesenchymal-like) (*p* = 0.07), compared to non-miliary P/M samples and compared to differences between A/S and P/M samples in miliary (Figures 2C and 2D).

### Differences between A/S and P/M samples

QuSAGE analysis using the EMT gene sets revealed decreased epithelial characteristics in A/S (*n* = 6) compared to P/M (*n* = 11) in non-miliary with unchanged mesenchymal characteristics (Figure 2D and Supplementary Figure S6B), indicating a transient EMT stage. In contrast, there was no significant difference in EMT characteristics between miliary A/S (*n* = 16) and P/M samples (*n* = 9; Figures 2C and 2D), which points towards different strategies to allow survival (and growth) of detached tumor cell in anaerobic ascites.

These differences in EMT characteristics are also evident from the number of significantly differentially expressed genes and deregulated pathways between A/S and P/M in the two spread types. In non-miliary, 1 537 genes were significantly differentially expressed and 23 pathways were deregulated between A/S and P/M compared to 597 genes and 8 pathways in miliary (Table 2, Folders ”nM_PM_AS”, “M_PM_AS” and Table “SignificantDiff_Genes.xlsx”). “Chemokine signaling” was by far the most activated pathway (FDR = 2.1e-13) in non-miliary, whereas “MicroRNAs in cancer” was the most activated pathway (FDR = 8.5e-08) in miliary with oncomirs, mir-221/mir-222, and mir-181 and -30e downregulated in ascites cells. Unsurprisingly, non-miliary A/S compared to P/M samples showed inhibited extracellular matrix (ECM)-interactions, activated Ras signaling and cytokine-cytokine receptor interaction pathways, and activation of a pathway associated with leukocyte transendothelial migration (Table 2).

Interestingly, non-coding genes were over-represented in A/S versus P/M in miliary compared to non-miliary, 34.6% compared to 25.1% (*p* < 0.001). The non-coding genes were annotated with the ten most significant GO-terms (NONCODE v4) and networks were built using these annotations (Supplementary Figure S3).

### TSF, EMF and overall survival

Microarray data of an independent cohort of 165 FIGO II/III/IV serous ovarian cancer patients [10] (GEO: GSE49997) were used to assess the impact of the tumor spread (TSF) and the epithelial-mesenchymal status of tumor cells (EMF) on patients, outcome using univariate and multiple Cox regression analyses (Table 3).

**Table 3:** Cox regression analysis of 165 FIGO II/III/IV serous ovarian cancer patients

Factor *n* = 165, 78 death	Univariate Cox regression		Multiple Cox regression	
	HR (CI_95_)	*p*	HR (CI_95_)	*p*
**Age (decades)**	**1.43 (1.16–1.75)**	**< 0.001**	**1.49 (1.20–1.86)**	**< 0.001**
**FIGO (IV vs III vs II)**	**2.51 (1.56–4.04)**	**< 0.001**	**2.69 (1.56–4.65)**	**< 0.001**
Grade (3 vs 1&2)	**2.11 (1.19–3.79)**	**0.011**	1.59 (0.87–2.94)	0.133
Residual tumor (yes vs no)	**1.76 (1.11–2.79)**	**0.017**	1.48 (0.91–2.40)	0.117
**Mol. Subclass (II vs I)**	**2.06 (1.29–3.28)**	**0.002**	**1.82 (1.11–2.98)**	**0.017**
**Peritoneal carc. (yes vs no)**	**3.72 (1.85–7.48)**	**< 0.001**	**2.73 (1.33–5.61)**	**0.006**
EMF	0.58 (0.31–1.08)	0.086	-^1^	-
**TSF**	1.86 (0.65–5.34)	0.247	**3.77 (1.14–12.39)**	**0.029**

1 Removed from the final Cox regression model by Akaike's information criterion selection (AIC, a variable-penalized criterion). If EMF is not excluded from the final model the TSF is even more predictive (HR = 4.24, CI_95_ (1.28–14.07), *p* = 0.018).

**Figure 4:**
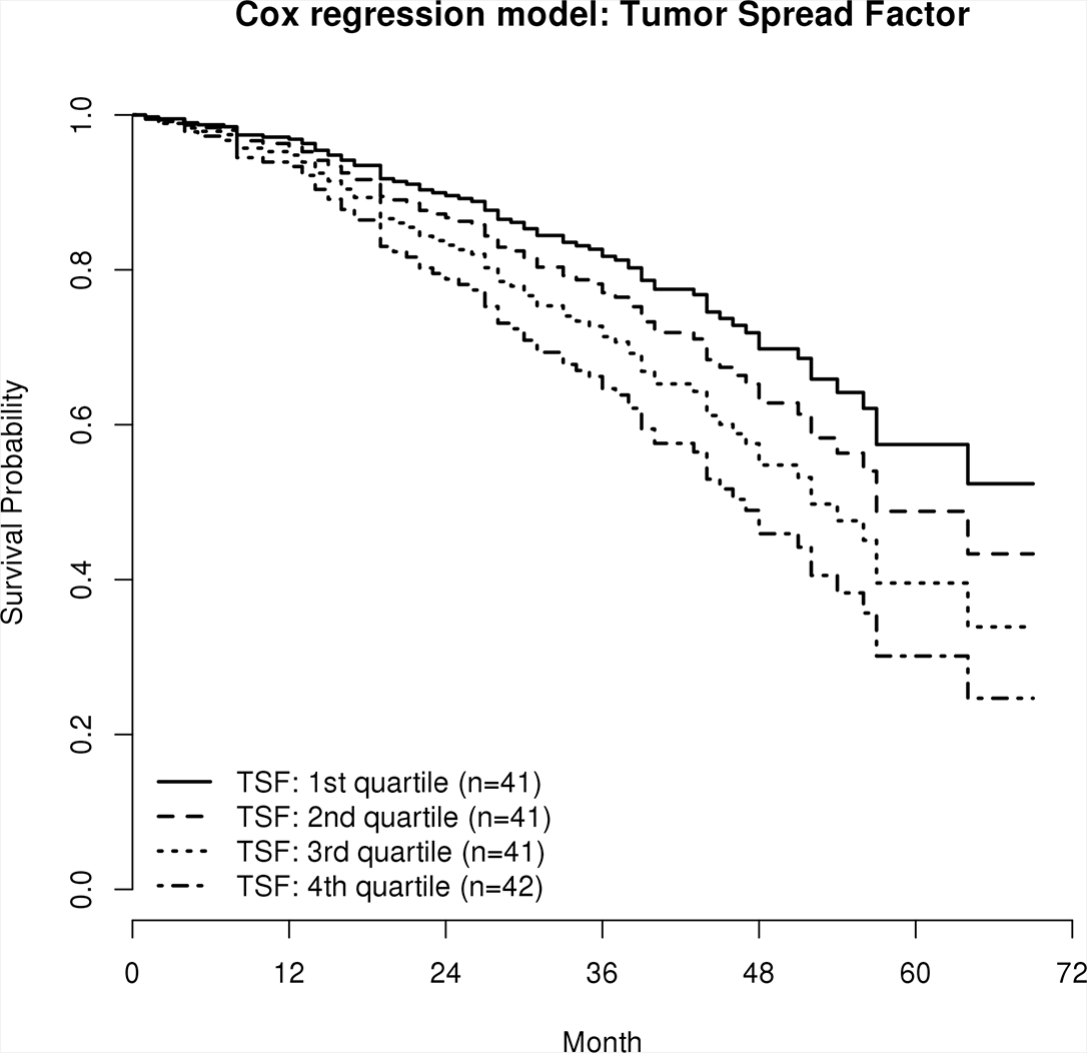
Survival estimates of the multiple Cox regression model (HR 3.77, *p* = 0.029; *cf*. Table 3) Patients were stratified according to the Tumor Spread Factor (TSF), calculated from the miliary and the non-miliary gene signatures in quartiles. Curves are corrected for all relevant clinicopathologic parameters and represent the survival estimates from the multiple Cox regression model, therefore no censored observations are indicated.

Interestingly, the TSF was a significant independent negative predictor for overall survival (OS) in multiple Cox regression analysis (HR = 3.77; CI_95_ 1.14–12.39; *p* = 0.028), which means that patients with a more miliary expression pattern have a worse prognosis, independent from age, FIGO stage, tumor grade, molecular subclass [10], peritoneal carcinomatosis, the EMF, and residual tumor mass after debulking surgery (Table 3). Figure 4 illustrates the impact of the TSF on overall survival corrected for all other clinicopathologic parameters (*cf*. Table 3) with survival curve estimates stratified by the TSF in quartiles. The TSF was not associated with any clinicopathologic parameter, not even with peritoneal carcinomatosis, but a weak, statistically not significant, negative association of TSF with distant metastases was observed. The univariate impact of TSF on OS was not significant (HR = 1.86; CI_95_ 0.65–5.34; *p* = 0.247).

The EMF showed a trend as univariate positive predictor for OS (HR = 0.58; CI_95_ 0.31–1.08; *p* = 0.086) which is in line with results from Miow *et al.* [16], but was excluded from the final multiple Cox regression model. Nevertheless, when the EMF was retained in the Cox regression model, the TSF showed an even stronger impact on OS (HR = 4.24, CI_95_ 1.28–14.07; *p* = 0.018).

## DISCUSSION

**Figure 5:**
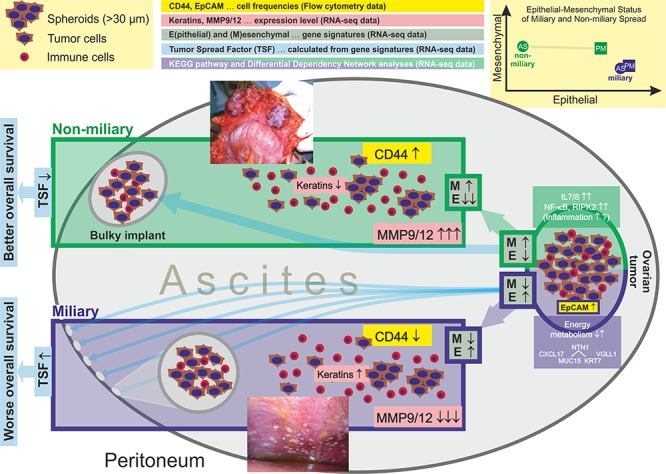
Summary of results shown in this publication (flow cytometry and transcriptomics) comparing cancer cells of patients with miliary and non-miliary peritoneal tumor spread *(image reprinted with permission from Medscape Reference* (http://emedicine.medscape.com/), *2014, available at*: http://emedicine.medscape.com/article/255771-overview). Non-miliary ascites samples showed more CD44+ cells, lower keratin expression, and higher MMP9/12 expression compared to miliary ascites samples. Ascites tumor cells of non-miliary patients showed a substantially reduced epithelial character compared to tumor cells from all other origins and tumor spread types. Ascites and solid tumor cells from non-miliary patients revealed globally increased mesenchymal characteristics compared to ascites and solid tumor cells from miliary patients.

We are the first to propose a new classification of high grade serous ovarian cancer patients on the basis of peritoneal tumor spread characteristics. We could show substantial molecular differences in the two apparent modes of peritoneal tumor spread, miliary versus non-miliary, and a strong negative impact of the miliary tumor spread on overall survival. This classification is supported by clinical observations, cell characteristics of ascites and solid tumor cells (flow cytometry), and transcriptome data (RNA-sequencing), indicating substantial biological differences between these two groups, summarized in Figure 5.

### Cell composition

Flow cytometric analysis revealed lower frequencies of CD44+ cells in ascites and a higher frequency of EpCAM+ cells in miliary ovarian tumors. CD44 is proposed to be a stemness marker in EOC [28] but also reactive mesothelial cells were shown to express this surface marker [29]. Reactive mesothelial cells are a response to inflammatory processes in a body cavity [30] and are (together with other evidences for a more inflamed situation in the non-miliary setting, e.g. higher IL7, IL8, NF-кB, and RIPK2 expression) the most probable explanation for the higher proportion of CD44+ cells in non-miliary ascites. The enrichment of EpCAM+ cells in miliary patients could be explained by either a higher tumor cell density (with less stromal and infiltrating immune cells) or a more epithelial and less mesenchymal characteristic of the tumor cells (see transcriptomics data). There was no difference in frequencies of EpCAM+ cells in peritoneal implants between miliary and non-miliary. Although differences in cell composition between patients with miliary and non-miliary tumor spread characteristics could be found in ascites and primary tumors, small patient numbers make it hard to assess the whole heterogeneity of these groups.

### Transcriptome analyses

In all performed comparisons (except for P versus M and A versus S) significant differences in gene expression were found, with highest numbers of differences comparing floating tumor cells with solid tumor masses in non-miliary patients (1 537 genes), followed by the same comparison in miliary patients (597 genes). Differences between miliary and non-miliary patients were substantially higher in floating tumor cells (215 genes) compared to only two genes in solid tumors. Differences in gene set expressions combined with the deregulated pathways found by PAGI analysis (mostly involved in cell metabolism) indicate, that miliary tumor cells seem to be more adapted to the anaerobic microenvironmental condition present in the peritoneal cavity and especially in malignant ascites. The large number of down-regulated gene sets in miliary P/M samples indicates a global reduction of transcriptional activity compared to P/M cells from non-miliary. There was no evidence for EMT between ascites and solid tumor cells in miliary, whereas the epithelial characteristics of ascites cells in the non-miliary subtype were greatly reduced compared to solid tumors (*cf*. Figure 5). Moreover, all non-miliary tumor tissues showed an enhanced mesenchymal status compared to miliary. Over-representation of downregulated non-coding genes together with “microRNAs in cancer” as the most significantly activated pathway between ascites and solid tumor cells in miliary, indicates a deregulated, i.e. under-represented, competing endogenous RNA (ceRNA) network. E.g. mir-30e, the most downregulated microRNA in ascites, is known to target ITGB3, which belongs to the integrin class of cell adhesion molecule receptors. This might lead to an upregulation of ITGB3 in miliary ascites tumor cells. Many downregulated protein-coding genes in miliary compared to non-miliary ascites cells were shown to be associated with cytokine-cytokine receptor interaction, chemokine signaling, ErbB signaling and estrogen signaling including PI3K and NF-κB. PI3K-driven NF-κB activation was shown to be associated with the promotion of expression and secretion of cytokines, thereby generating a pro-tumor microenvironment [31], e.g. by increasing the activation of stromal cells. This explains the increased frequency of CD44+ cells in non-miliary ascites. CD44 is an adhesion protein, capable of binding to the ECM and a proposed stem cell marker in several types of cancers [32]. Besides CD44 expression on reactive mesothelial cells, CD44 was shown to be expressed on cancer associated fibroblasts (CAF), especially in hypoxic areas (like ascites). *In vitro*, CAFs from wild-type mice could be shown to sustain the stemness of cancer stem cells [33]. Matrix metalloproteinases MMP9 and MMP12 were among the most downregulated genes in miliary compared to non-miliary ascites. Usually, upregulation of matrix metalloproteases is an important event in tumor invasion and metastasis, as they are known to mediate degradation of the ECM. Contrary to our results, high *MMP9* expression was shown to correlate with poor prognosis in EOC [34]. The most upregulated gene in miliary was keratin 4, a member of the type II keratin gene family. Keratins are associated with epithelial cell adhesion by stabilizing desmosomes, as shown in keratinocytes [35]. Summarized, the miliary implantation pattern in HGSOC does not follow usual metastatic routes, but rather new implantation mechanisms for the colonization of a surface with small, nodular lesions has to be proposed.

In conclusion, there seem to be two different mechanisms of how tumor cells of HGSOC patients populate ascites, a) in non-miliary by reducing the epithelial characteristics of the tumor cells, leading to cells which are less capable of implanting on the peritoneal surface, but apart from that induce more inflammation (as indicated by higher frequencies of CD44+/EpCAM-, putative reactive mesothelial cells, NF-κB, and IL7/8), or b) in miliary by globally reducing cell metabolism and the ceRNA network, remodeling of energy pathways and allowing survival in anaerobic conditions, but keeping epithelial characteristics and thus being more capable of adhering to and implanting the peritoneal wall. The non-miliary type is characterized by globally more mesenchymal and less epithelial gene expression characteristics, already manifested in the solid ovarian tumor mass (Figure 5). This raises the question, whether all cells in the tumor are uniformly more mesenchymal and less epithelial-like in non-miliary or if a subfraction of cells gained mesenchymal characteristics, making it possible to leave the solid tumor tissue and to populate the ascites (our gene expression data cannot discriminate between these two scenarios). Conceivably, tumor cells promote a miliary tumor spread by cell-cell adhesion between tumor cells forming spheroids rather than single cells adhering to and invading the ECM underlying the mesothelium that lines the peritoneal cavity. These tumor cell spheroids adhere to the peritoneum as whole aggregates, forming the characteristic small nodules in miliary. This was shown recently in a mouse model, where cultured spheroids were injected into the peritoneal cavity of nude mice which resulted in adhesion of tumor cells to kidney and ovary but without obvious invasion of the organs [36]. Our data support these findings.

### Overall survival

The clinical relevance of the tumor spread pattern (TSF) and the epithelial-mesenchymal status (EMF) were assessed by multiple Cox regression analysis with an independent cohort of serous FIGO II/III/IV EOC patients. Miliary (high TSF) proofed to be a significant and independent negative predictor for overall survival. In EOC, more epithelial characteristics of cancer cells seem to correlate with better prognosis [15], explainable by the assumption that mesenchymal characteristics are needed for distant metastasis (via lymph or blood circulation). This is in line with the trend towards better prognosis for high EMF as observed in our data. Interestingly, there is a weak but significant positive correlation of TSF and EMF, whereas the impact on survival is reverse. This indicates that TSF has a stronger prognostic impact than EMF.

### Outlook and clinical relevance

The evaluation of the tumor spread pattern could aid in treatment decision making. To date, Catumaxomab (Removab^®^) a trifunctional anti-EpCAM-anti-CD3 antibody [37] is already used as second line therapy for malignant ascites in several tumor entities but with severe side effects [38]. Given i) the negative impact of miliary tumor spread ii) the less inflamed situation in the peritoneal cavity in patients with miliary tumor spread and iii) the fact that tumor cells from miliary patients are more epithelial like, such patients could profit from a Catumaxomab therapy already in first line.

### Shortcomings

All data about deregulated genes, gene sets, and pathways must be regarded as intergroup differences between samples from patients with miliary and non-miliary tumor spread or between ascites and solid tumor cells, since we did not include normal tissues from healthy controls (reason: no clear origin of HGSOC, fallopian tube or ovarian surface and unavailability of such cells in necessary amount and purity). Another critical point was the enrichment of tumor cells for RNA-sequencing by EpCAM: on the one hand important for direct comparability of results (removing the largely different tumor microenvironments in solid tumors and ascites), but on the other hand introducing a bias towards epithelial tumor cells, with the consequence, that mesenchymal tumor cells which completely lost their EpCAM expression were not co-enriched. Finally, following our concept of analyzing a small but rigorously selected and well documented number of patients with comprehensive explorative methods (including gene-set, pathway, and network analyses) will find large biological differences but probably not cover the complete biological heterogeneity in each group.

## CONCLUSION

We conclude that the mode of peritoneal tumor spread, either miliary or non-miliary, which we describe and introduce in this work, is independent from other classification approaches (especially the one described by Yoshihara *et al*. [8] and validated by us [10]) and even independent from the concept of peritoneal carcinomatosis. Major differences of tumor cells, especially in the ascites, between non-miliary and miliary patients are less epithelial characteristics and (consistently) a more active inflammatory status in non-miliary. Whereas in other cancer entities less epithelial characteristics are usually associated with worse prognosis, the impact on overall survival is clearly contrary in our study. This is in concordance with the typical course of this disease: intraperitoneal spread and less prominent lymphatic and even less vascular metastasizing. Therefore characteristics supporting such local recurrences, i.e. more epithelial like and thus implantation prone tumor cells in the ascites, are negative predictors for survival and renders ovarian cancer a special case compared to other carcinomas where distant metastases (triggered by EMT-MET of tumor cells) are the major complications. This should be taken into account when searching for new therapeutic strategies and interpreting and transferring data obtained in other cancer entities to HGSOC.

## MATERIALS AND METHODS

### Study design

Tumor tissues from ovarian and peritoneal origin and ascites of chemotherapy-naive patients with HGSOC were collected between March 2012 and May 2013 at the Medical University of Vienna. All patients signed an informed consent and approval for this study was obtained by the ethical review board (no. 793/2011). Peritoneal tumor spread was categorized during primary surgery in miliary and non-miliary (Table 1). Clinicopathological characteristics including histology, FIGO stage, and grade were assessed by a pathologist specialized for gynecological malignancies (Supplementary Table 1).

### Preparation of patient material

#### Ascites

Fresh ascites was filtered consecutively for tumor cell aggregates (spheroids) using *CellTrics*^®^ filters with mesh sizes of 150 μm, 30 μm, and 20 μm. Spheroids were isolated from the washed 30 μm-filter and single cells were isolated from the 20 μm filtrate. Aliquots from both fractions were cryopreserved in liquid nitrogen with 5% DMSO in cell free ascites. For gene expression analysis, EpCAM+ cells were isolated from the 20 μm filtrate with Dynabeads Epithelial Enrich (Invitrogen, Carlsbad, CA, USA) and a magnetic bead-based cell separation device. The ascites EpCAM+ single cells and the ascites spheroids were lysed with QIAzol and stored at −80°C for subsequent RNA preparation. For flow cytometric analyses spheroids were disaggregated enzymatically (Accutase^®^ Cell Dissociation Reagent, Life Technologies, 37°C, 10 min, 0.5 mL) and mechanically (repeated pipetting, 50 times). Subsequently, cells were incubated in growth medium (DMEM, 10% FCS) for 30 minutes at 37°C to allow re-expression of surface markers.

#### Tumor tissue

Tissue slices of ovarian and peritoneal tumor masses were obtained during surgery, minced, and digested with 1.04 U ml^−1^ Liberase DH research grade (Roche, Basel, Switzerland) while stirring at 37°C for one hour. A representative piece of this tissue was fixed, paraffin embedded, and a hematoxylin-eosin stained section was examined by the pathologist. The cell suspension was supplemented with 4 mM EDTA and depleted of undigested tissues using a 40 μm cell strainer. The filtrate was used for enrichment of EpCAM+ cells as described above. Similarly, CD45+ cells were isolated and the fraction of CD45-depleted cells was cryopreserved for subsequent flow cytometric analyses of tumor cells.

#### RNA preparation

Total RNA from QIAzol lysed cell samples was isolated using the miRNeasy^®^ Mini Kit (Qiagen, Hilden, Germany) and concentration quantified using a NanoDrop ND-1000 spectrophotometer (Thermo Fisher Scientific, Waltham, USA) and RiboGreen RNA Reagent (Invitrogen, Carlsbad, CA, USA). Quality was assessed by an Agilent 2100 Bioanalyzer (Santa Clara, CA, USA) and only samples with an RNA Integrity Number (RIN) >7.7 were used for library preparation.

#### Mutation analysis

TP53 mutations were assessed by a functional assay (FASAY) [39] and confirmed by Sanger sequencing.

### Flow cytometry (FC)

Following fluorophore-conjugated antibodies were used for flow cytometry: FITC mouse anti-human CD45, (clone HI30, BD Bioscience, NJ, USA), PerCP-eFluor 710 rat anti-human CD326 (clone 1B7, eBioscience, San Diego, CA, USA), PE-Cy7 rat anti-CD44 (clone IM7, eBioscience, San Diego, CA, USA) phycoerythrin (PE) mouse anti-L1CAM (clone 5G3, abcam, Cambridge, UK), and allophycocyanin (APC) mouse anti-CD133 (clone AC133, Miltenyi Biotec, Bergisch Gladbach, Germany). Samples were stained for dead cells using LIVE/DEAD^®^ Fixable Dead Cell Stain (Invitrogen™ Carlsbad, CA, USA) according to the manufacturer's protocol, followed by fixation in 1% formaldehyde. Subsequently, cells were stained with the antibody-mix. All samples were measured on a BD LSRFortessa flow cytometer, equipped with three lasers (405 nm, 488 nm, and 640 nm). Analysis was performed using FlowJo software (v7.6.2, Tree Star, Inc., Ashland, OR, USA). Dead and CD45+ cells were excluded from further analysis and all combinations of positive cells were systematically assessed using the same gating strategy (see Supplementary Figure S1).

### Statistical analysis

Statistical analysis was performed with R (v3.1.2) [40]. Mann-Whitney-U test and Kruskal-Wallis test were used for two-group and multi-group comparisons. Two-sided *p*-values below 0.05 were considered as significant. Principal Component Analysis of centered and scaled data was used to identify associations between flow cytometry data and clinical parameters.

### Library preparation, RNA sequencing, and bioinformatic analyses

For details see Supplementary methods.

## SUPPLEMENTARY INFORMATION FIGURES AND TABLES


