# Supplementary material for: Peritoneal tumor spread in serous ovarian cancer-epithelial mesenchymal status and outcome
Source: Oncotarget. 2015 May 11;6(19):17261–75. doi: 10.18632/oncotarget.3746 (PMC4627306; doi:10.18632/oncotarget.3746)
Supplement: Supplementary file 1 [file oncotarget-06-17261-s001.pdf]

SUPPLEMENTARY INFORMATION

SUPPLEMENTARY METHODS

Flow cytometry – gating strategy

A comprehensive gating strategy was employed on data retrieved from flow cytometric analyses of ascites and tumor tissues on a BD LSRFortessa™ cell analyzer (BD Biosciences, Franklin Lakes, New Jersey, USA) equipped with three lasers (405 nm, 488 nm, and 640 nm) and FACSDiVA software (BD, New Jersey, USA).

Digital files from around 20,000 events were exported in fcs 3.0 format and gating was performed using FlowJo software (v7.6.2). Prior to analyzing actual patients' material a gating template was set up using PBMCs and test ascites to ensure a uniform analysis of all samples.

Firstly, the flow cytometry data set of each individual patient (comprising data from ascites single cells, spheroids, primary and peritoneal tumor tissue) was visually assessed in the forward scatter (FSC)–side scatter (SSC) plot (Fig. S1A). Secondly, cell doublets and triplets were discriminated in the SSC-area (SSC-A) and FSC-width blot (FSC-W) (Fig. S1B). To ensure the counting of whole cells and to gate out cell debris a cut-off at 400 in the FSC channel was employed (Fig. S1C). Events from the single cell gate without debris were then checked for viability in the SSC-A and Alexa-405-A fluorescence channel (emission of live/dead stain, Fig. S1D). Furthermore, immune cells were discriminated by gating out CD45 positive events, visible in the FITC channel (Fig. S1E). In the PerCP-eFluor710 channel (Fig. S1F) cells were sorted in EpCAM positive and negative events, for CD44 positivity in the PE-Cy7 channel (Fig. S12H), for L1CAM positivity in the PE channel (Fig. S1G) and for CD133 positivity in the APC channel (Fig. S1I). Furthermore, EpCAM+ (Fig. S1J) and EpCAM- (Fig. S1K) events were gated for combined L1CAM/CD44, L1CAM/CD133, and CD44/CD133 double positivity. The resulting frequencies of populations (in % of living/CD45- events) were collected in a table (*cf*. Tab. S2).

RNA-sequencing and bioinformatics

Sequencing libraries were prepared from 200 ng total RNA isolated with the miRNeasy Mini Kit (Qiagen, Hilden, Germany). RNA quality was controlled on a RNA Pico Chip (Bioanalyzer 2100, Agilent, Santa Clara, CA, USA) and quantified with NanoDrop ND-1000 spectrophotometer (Thermo Fisher Scientific, Waltham, USA) and RiboGreen RNA Reagent (Invitrogen, Carlsbad, CA, USA). Ribosomal RNAs were removed using the Ribo-Zero™ rRNA Removal Kit (Epicentre, Madison, WI, USA) and libraries prepared employing the NEXTflex RNA-Seq and Barcoding Kits (Bioo Scientific, Austin, TX, USA) with halved reaction volumes with an epMotion 5075 automated pipetting system (Eppendorf, Hamburg, Germany) according to the manufactures instructions. Quality of libraries was assessed on a DNA High Sensitivity chip (Bioanalyzer 2100) and quantified with a digital PCR system (QX100™ Droplet Digital™ PCR System, BioRad, Hercules, CA, USA) and the ddPC Library Quantification Kit (BioRad). Eight libraries were pooled equimolarly and sequenced for 50 bp paired ends on an Illumina HiSeq 2000 system (San Diego, CA, USA). After demultiplexing of the reads the quality of the libraries was checked with FastQC (http://www.bioinformatics.babraham.ac.uk/projects/fastqc/) and RSeQC (1) and the paired end reads mapped to the human genome and transcriptome (HG19) with the RNA Seq Unified Mapper RUM v2.0.4 (2). To filter out all reads belonging to putative circular RNAs, which would compromise differential gene expression analysis because of their higher stability, the circularization coordinates for each circular RNA were predicted with scripts from the Rajewsky group (http://circrna.org 2013/06/12, (3)) and all read pairs were removed from the SAM-files which mapped completely inside the circularization coordinates (4). Filtered SAM-files were finally used for counting in fragments into the gene model from the GENCODE Project (version 18, http://www.gencodegenes.org/) using htseq-count (HTSeq v0.5.4p2, http://www-huber.embl.de/users/anders/HTSeq, (5)) with option "union". Differential gene expression was analyzed with R-package limma 3.16.6 (6) using sample type (A, S, P, or M) and mode of tumor spread as variables for the design matrix. Raw read counts were loaded using limma's voom-function, linear models fitted for each gene using the design matrix, and differentially expressed genes called using an empirical Bayes statistics (eBayes-function). False discovery rates (FDR) were estimated with Benjamini and Hochberg's method. Following comparisons (contrasts) were calculated: miliar tumor spread versus non-miliar tumor spread in all samples, in A/S, and in P/M. Also, A/S and P/M were compared in the non-miliary and in the miliary subset of samples. For the latter two a FDR cut-off of 5% and for the former a 10% cut-off was employed (see Table "SignificantDiff_Genes.xlsx" in the supplementary data file).

Dimensionality reduction of normalized $\log_2$ gene expression values was performed with the non-linear dimensionality reduction method Isomap (7) implemented in the R-package RDRToolbox 1.10.0. Three dimensions were calculated with the modified version of the original Isomap algorithm including the nearest and farthest 21 neighbors and the first two dimensions are shown in a plot (dimension 1 versus dimension 2, Supplementary Figure S2).

The Differential Dependency Pathway between miliary and non-miliary samples was constructed with

the KDDN plugin v1.1.0 (8) for Cytoscape 3.2.0 (9) using default parameters and 1 000 permutations. Disturbed KEGG pathways were assessed using significant genes with their $\log_2$ fold changes by Signaling Pathway Impact Analysis (SPIA v2.18.0 (10)) and significant KEGG pathways were illustrated by R-package pathview v1.6.0 (11). For the comparison of miliary versus non-miliary in P/M samples (only two genes significant) the more sensitive method PAGI v1.0, based on global influence from the internal effect of pathways and crosstalk between pathways, was used. In Supplementary Figure S4 global influence factors (GIFs) of the highest impact genes are shown. Gene set analysis using the Molecular Signatures Database (MSigDB) v4.0 (12) and the epithelial and mesenchymal gene sets (13) was performed with the Quantitative Set Analysis for Gene Expression (QuSAGE) method (14). Gene sets for miliary (110 genes) and non-miliary (162 genes) were defined from P/M samples by the R-package CellMix v1.6.2 (15) using the method *abbas* with gene expression values averaged over gene symbols and a threshold of 0.01 (Table "Spread_gene_signatures.xlsx" in the supplementary data file). Annotation of both gene sets was performed with the disease ontology database (DO terms, (16)) and illustrated with the R-package DOSE v2.4.0. Finally, the R-package dnet v1.0.6 (17) was used to find the high-scoring networks for each comparison, either using the STRING v9.1 database of known and predicted protein interactions or the more selective CCSB Human Interactome database (HI-III, preliminary release 1.3, downloaded from http://interactome.dfci.harvard.edu) of only experimentally verified protein-protein interactions.

Annotation of differentially expressed non-coding genes was performed with the NONCODE v4 database (18). Briefly, non-coding genes were mapped to the non-coding identifier of the NONCODE v4 database and each annotable non-coding gene was connected to the ten most significant GO-ontology terms (file "NONCODEv4_human.func.gz" from the download-site of http://www.bioinfo.org/noncode). Finally, a network from all of these edges was built and all nodes with less than four edges were removed. The remaining network was plotted and the nodes were color coded according to the $\log_2$ fold-changes between the respective comparisons. Circular nodes represent non-coding genes and grey square nodes GO terms (Figure S3).

Survival analysis

Gene expression data and accompanied clinical information were the same as used in Pils *et al*. (19), downloadable from the Gene Expression Omnibus repository, GSE49997. The Tumor Spread Factor (TSF) and the Epithelial Mesenchymal Factor (EMF) were calculated from normalized gene expression data as outlined in the main manuscript (164 genes out of 272 could be mapped for the TSF and 283 genes out of 308 for the EMF). Survival analysis was performed in R and Cox regression model selection was done by minimizing the Akaike information criterion (AIC) by the stepAIC function from the MASS R-package.

REFERENCES

1. Wang L, Wang S, Li W. RSeQC: quality control of RNA-seq experiments. Bioinformatics. 2012; 28:2184–5.
2. Grant GR, Farkas MH, Pizarro AD, Lahens NF, Schug J, Brunk BP, et al. Comparative analysis of RNA-Seq alignment algorithms and the RNA-Seq unified mapper (RUM). Bioinformatics. 2011; 27:2518–28.
3. Memczak S, Jens M, Elefsinioti A, Torti F, Krueger J, Rybak A, et al. Circular RNAs are a large class of animal RNAs with regulatory potency. Nature. 2013; 495:333–8.
4. Bachmayr-Heyda A, Reiner AT, Auer K, Sukhbaatar N, Aust S, Bachleitner-Hofmann T, et al. Correlation of circular RNA abundance with proliferation - exemplified with colorectal and ovarian cancer, idiopathic lung fibrosis, and normal human tissues. Scientific reports. 2015; 5:8057.
5. Anders S, Pyl PT, Huber W. HTSeq-a Python framework to work with high-throughput sequencing data. Bioinformatics. 2015; 31:166–9.
6. Smyth GK. limma: Linear Models for Microarray Data. Gentleman R, Carey V, Huber W, Irizarry R, Dudoit S. Bioinformatics and Computational Biology Solutions Using R and Bioconductor. Statistics for Biology and HealthSpringer New York: 2005; :397–420.
7. Tenenbaum JB, de Silva V, Langford JC. A global geometric framework for nonlinear dimensionality reduction. Science. 2000; 290:2319–23.
8. Tian Y, Zhang B, Hoffman EP, Clarke R, Zhang Z, Shih Ie M, et al. KDDN: an open-source Cytoscape app for constructing differential dependency networks with significant rewiring. Bioinformatics. 2015; 31:287–9.
9. Smoot ME, Ono K, Ruscheinski J, Wang PL, Ideker T. Cytoscape 2(8), new features for data integration and network visualization. Bioinformatics. 2011; 27:431–2.
10. Tarca AL, Draghici S, Khatri P, Hassan SS, Mittal P, Kim JS, et al. A novel signaling pathway impact analysis. Bioinformatics. 2009; 25:75–82.
11. Luo W, Brouwer C. Pathview: an R/Bioconductor package for pathway-based data integration and visualization. Bioinformatics. 2013; 29:1830–1.
12. Subramanian A, Tamayo P, Mootha VK, Mukherjee S, Ebert BL, Gillette MA, et al. Gene set enrichment analysis: a knowledge-based approach for interpreting genome-wide expression profiles. Proc Natl Acad Sci U S A. 2005; 102:15545–50.

13. Miow QH, Tan TZ, Ye J, Lau JA, Yokomizo T, Thiery JP, et al. Epithelial-mesenchymal status renders differential responses to cisplatin in ovarian cancer. Oncogene. 2014; .
14. Yaari G, Bolen CR, Thakar J, Kleinstein SH. Quantitative set analysis for gene expression: a method to quantify gene set differential expression including gene-gene correlations. Nucleic acids research. 2013; 41:e170.
15. Gaujoux R, Seoighe C. CellMix: a comprehensive toolbox for gene expression deconvolution. Bioinformatics. 2013; 29:2211–2.
16. Kibbe WA, Arze C, Felix V, Mitraka E, Bolton E, Fu G, et al. Disease Ontology 2015 update: an expanded and updated database of human diseases for linking biomedical knowledge through disease data. Nucleic acids research. 2015; 43:D1071–8.
17. Fang H, Gough J. The ‘dnet’ approach promotes emerging research on cancer patient survival. Genome medicine. 2014; 6:64.
18. Xie C, Yuan J, Li H, Li M, Zhao G, Bu D, et al. NONCODEv4: exploring the world of long non-coding RNA genes. Nucleic acids research. 2014; 42:D98–103.
19. Pils D, Hager G, Tong D, Aust S, Heinze G, Kohl M, et al. Validating the impact of a molecular subtype in ovarian cancer on outcomes: a study of the OVCAD Consortium. Cancer Sci. 2012; 103:1334–41.

SUPPLEMENTARY FIGURES AND TABLES

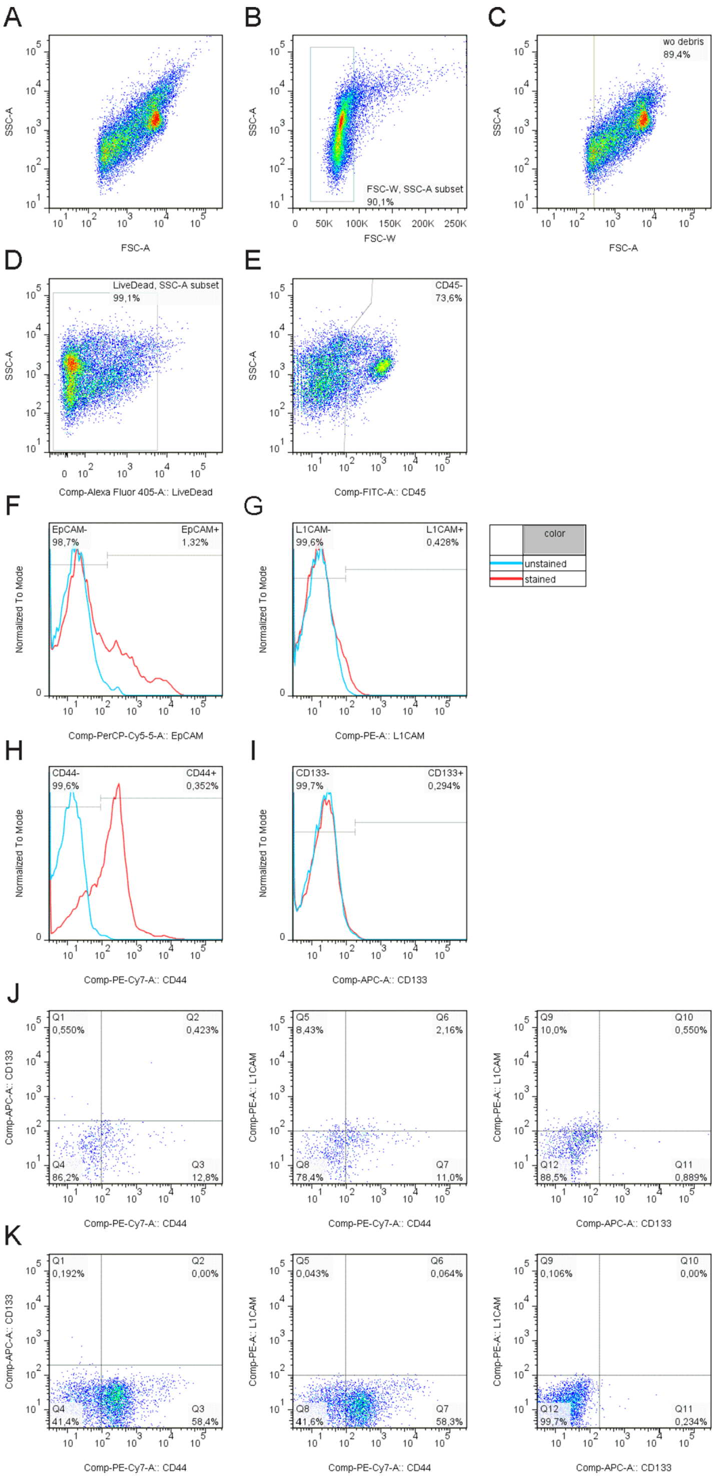

Supplementary Figure S1: Gating strategy for flow cytometric analysis. The chronological sequential of gates is resembled by the alphabetical order of the letters. B-E Each following plot is constructed from the gated population of the plot before. F-I Gating of CD45- populations. J Gating of CD45- EpCAM+ populations, K gating of CD45-EpCAM- populations.

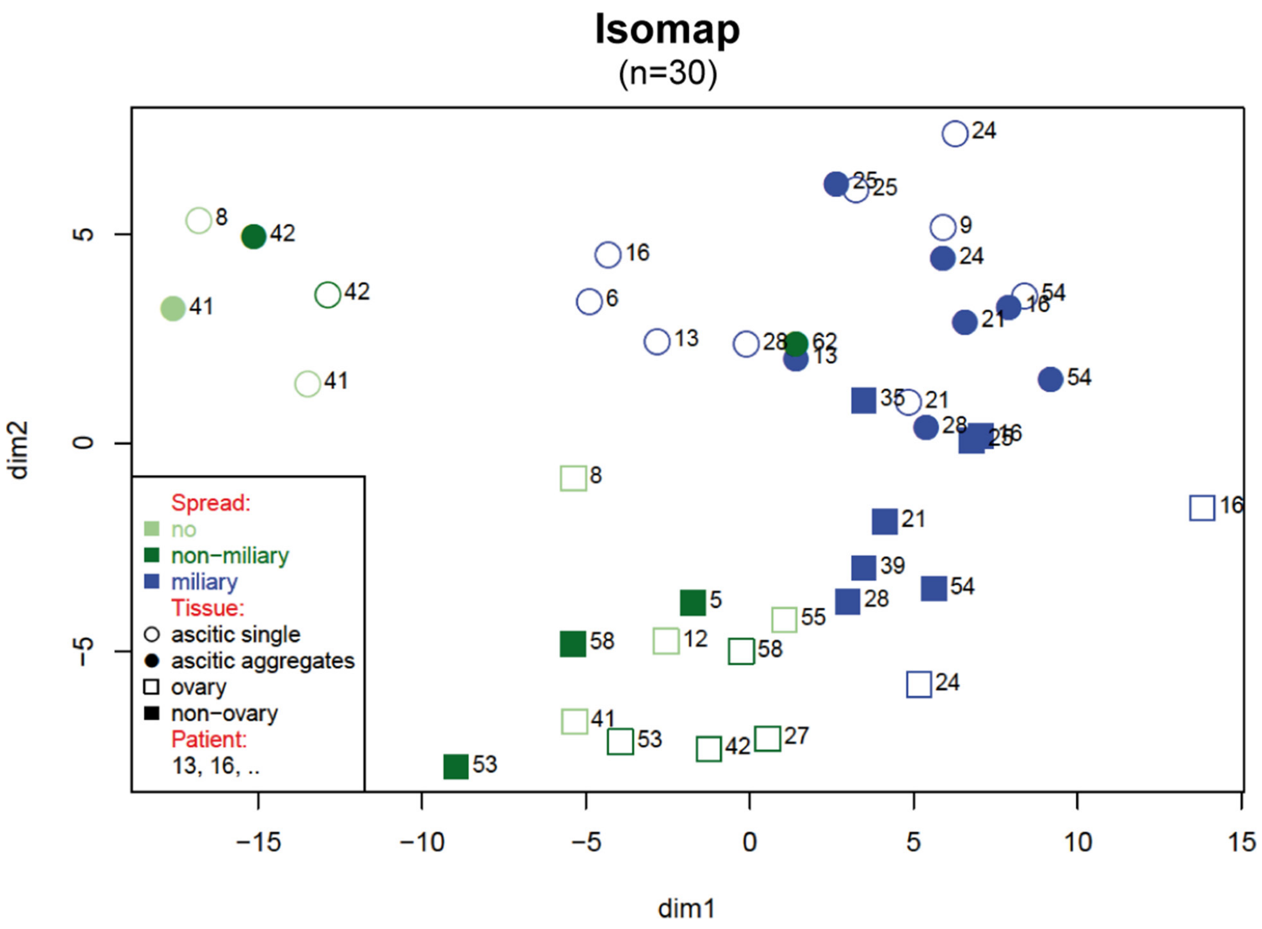

Supplementary Figure S2: Isomap. of all samples using differentially expressed genes between miliary and non-miliary (FDR 5%).

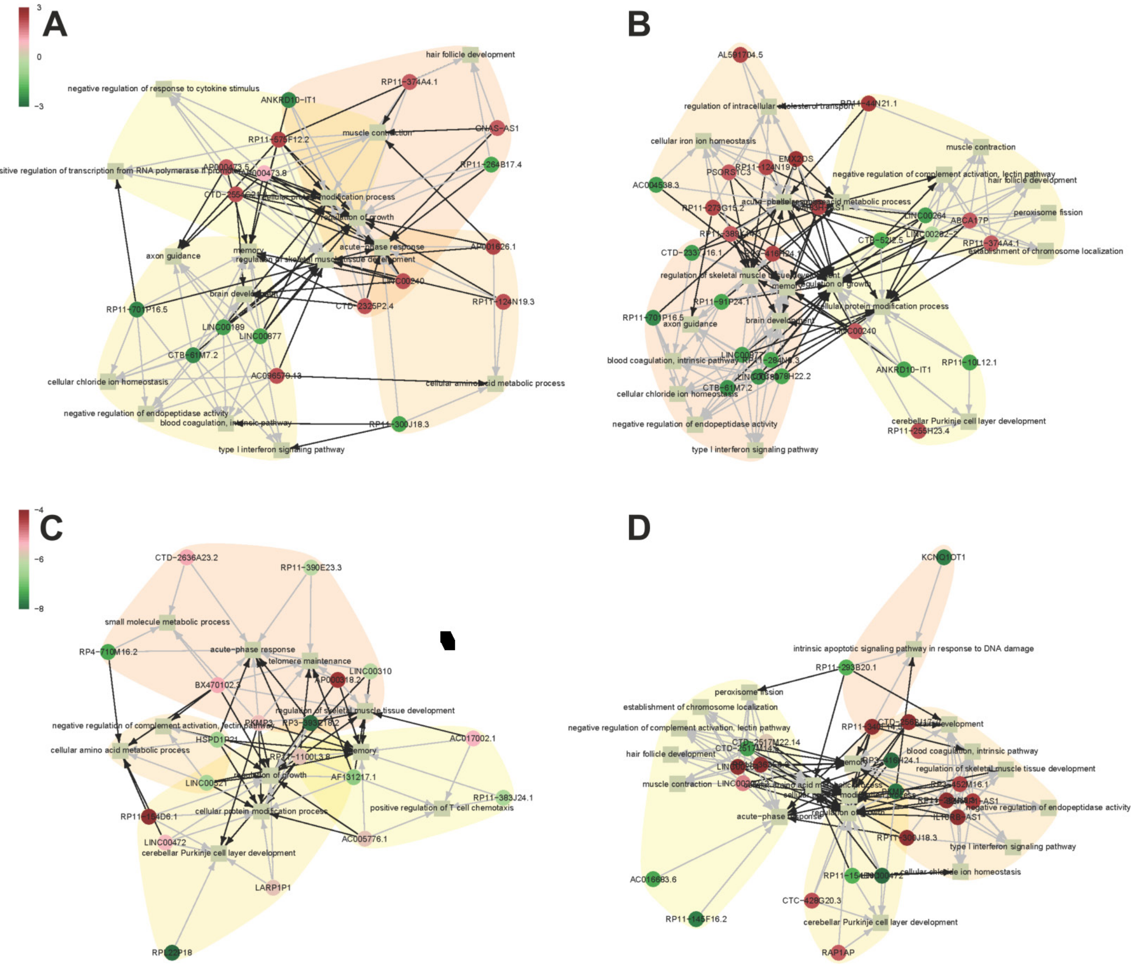

Supplementary Figure S3: Gene ontology (GO) annotation network. of significantly differentially expressed non-coding genes from comparisons miliary versus non-miliary in **A.** all samples, **B.** in AS samples, AS versus PM in **C.** miliary, and in **D.**) non-miliary samples. Circle nodes represent non-coding genes, colored according to $\log_2$ fold-changes in the respective comparison (red, up in miliary or AS). Square nodes represent connected GO terms (CAVE: in comparison AS versus PM in miliary all genes were downregulated in the former, therefore the color code represents only down-regulated fold-changes between -4 (red) and -8 (green)).

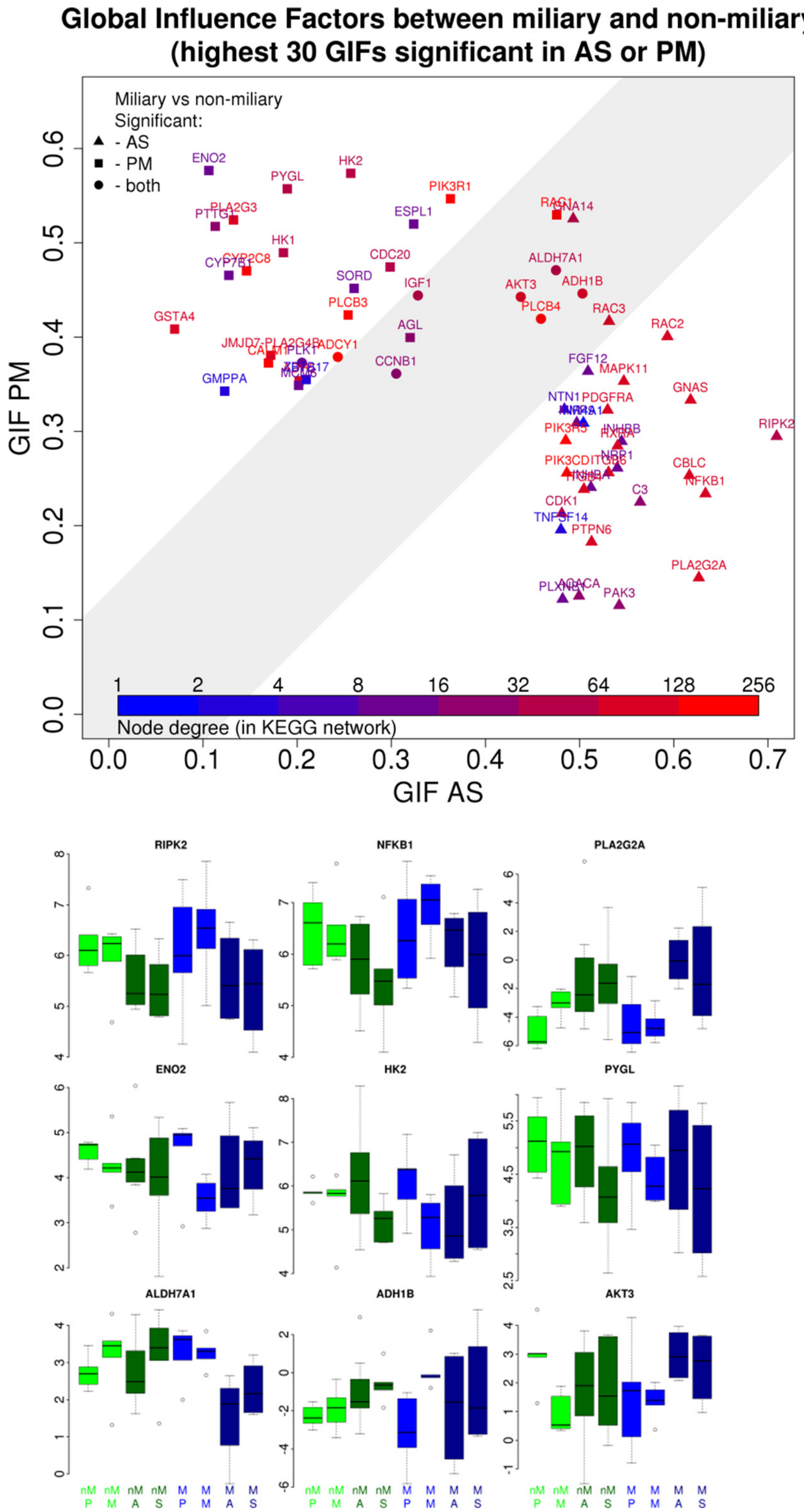

Supplementary Figure S4: PAGI analysis. showing the global influence factors (GIF) of each of the 30 highest genes from comparisons miliary versus non-miliary in AS and PM samples (upper plot) and boxplots of the gene expressions of six of the genes with the highest GIF values in one of the comparisons (lower panel).

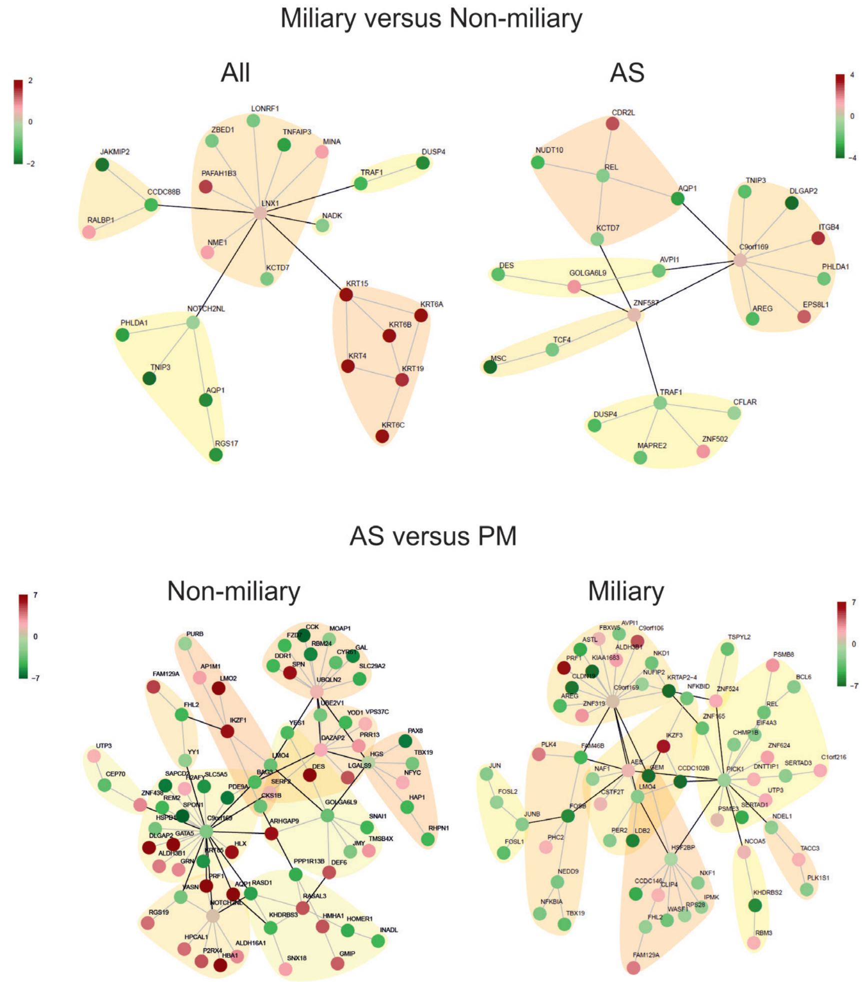

Supplementary Figure S5: High-scoring protein-protein interaction (PPI) networks. using only experimentally validated PPIs. Colors correspond to $\log_2$ fold-changes in the comparison (red, up in miliary mode of tumor spread for upper panel and up in AS for lower panel).

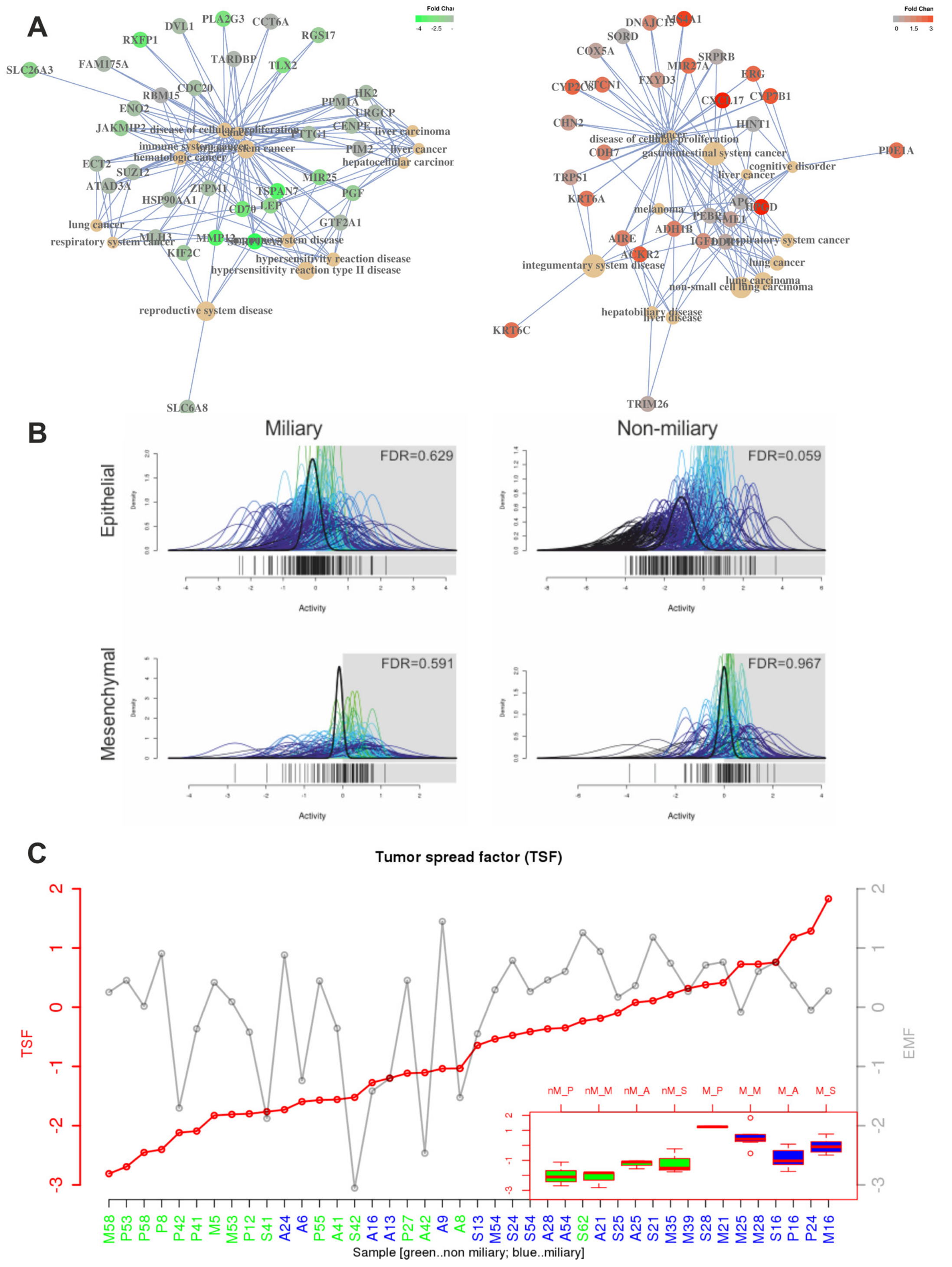

Supplementary Figure S6: A. Disease ontology (DO) network of the protein coding miliary gene set (left) and the non-miliary gene set (right). Colors correspond to fold changes between miliary (red, up in miliary) and non-miliary samples (green, up in non-miliary). **B.** EMF gene set analysis (qusage) showing differences in the epithelial (upper panel) and the mesenchymal (lower panel) gene sets between all AS and PM samples from patients with either miliary or non-miliary tumor spread. The x-axis ("Activity") represents probability density functions (pdf) of fold-changes of every gene in the gene-set (colored) between the corresponding comparison and a combined probability density function over all genes in the gene-set (bold black). A combined pdf around 0 indicates no change in the activity of the gene set in the corresponding comparison. **C.** Comparison between Tumor spread factor (TSF, red, left y-axis) and Epithelial mesenchymal factor (EMF, grey, right y-axis) in all analyzed patients' samples. Boxplots showing the TSF in different tissues (P, M, A, and S) separated by miliary (M, green) and non-miliary (nM, blue).

Supplementary Table S1: Patients enrolled in the study and performed analyses

Patient	Age	FIGO	Grade	Lymph nodes	Spread[1]	Ascites (ml)	Mutation	RNA-seq[2]	FC[2]	Residual Tumor[3]
5	80	IIIC	G2	pN1	1	< 500	TP53	M	-	1
6	70	IIIC	G2	pNX	2	>500	TP53	A	-	1
8	53	IIIC	G2	pN1	0	0	TP53	A, P	-	0
9	62	IIIC	G2	pNX	2	>500	TP53	A	-	1
12	55	IIIC	G3	pN1	0	>500	TP53	P	A, P, M	0
13	60	IIIC	G3	pN1	2	>500	TP53	A, S	A, S	1
16	64	IIIC	G3	pNX	2	>500	TP53	A, S, P, M	A, S, P, M	1
21	68	IIIC	G3	pNX	2	>500	TP53	A, S, M	A, S, M	0
24	50	IIIB	G2	pNX	2	>500	TP53	A, S, P	A, S, P, M	1
25	50	IIIC	G3	pN1	2	>500	TP53	A, S, M	A, S, P, M	0
27	66	IIIA	G3	pN0	1	0	TP53	P	P	0
28	57	IIIC	G3	pNX	2	< 500	TP53	A, S, M	A, S, M	0
30	41	IIIC	G3	pN0	1	< 500	TP53	-	P, M	0
31	69	IIIB	G3	pNX	2	< 500	n.d.[4]	-	M	0
35	56	IIIB	G3	pNX	2	0	TP53	M	M	0
39	50	IIIC	G3	pN0	2	0	TP53	M	-	0
41	49	IIC	G3	pN0	0	>500	TP53	A, S, P	A, S, P	0
42	49	IIIB	G3	pN0	1	>500	TP53	A, S, P	A, S	0
53	48	IIIC	G3	pN1	1	0	TP53	P, M	P, M	0
54	34	IV	G3	pNX	2	>500	TP53	A, S, M	A, S, M	1
55	81	IIa	G3	pN0	0	0	TP53	P	-	0
58	66	IIIC	G3	pNX	1	0	TP53	P, M	-	0
62	54	IIIC	G3	pN0	1	< 500	TP53	S	-	0

[1] Code as defined in Table 1.
[2] A, ascites single cells; S, ascites aggregated cells ("spheroids"); P, solid ovarian tumor mass ("primary tumor"); M, solid peritoneal tumor mass ("metastasis").
[3] 0, no residual tumor; 1, macroscopic residual tumor.
[4] n.d., not determined.

Supplementary Table S2: Gated cell populations and frequency-dependencies. Freq., frequency.

Population	Name in Graphs	Statistics		
Living cells	alive	*100%*		
CD45+	C45p	Freq. of alive	100%	
CD45-	C45n	Freq. of alive		
	CD44+	C44pC45n	Freq. of alive*	
CD45-	CD133+	C133pC45n	Freq. of alive*	
	L1CAM+	L1pC45n	Freq. of alive*	
	EpCAM+	EpC45n	Freq. of alive*	
		CD44-CD133+	EpC133pC44n	Freq. of CD45-
		CD44+CD133+	EpC133pC44p	Freq. of CD45-
		CD44+CD133-	EpC133nC44p	Freq. of CD45-
		CD44-CD133-	EpC133nC44n	Freq. of CD45-
		CD44-L1CAM+	EpL1pC44n	Freq. of CD45-
		CD44+L1CAM+	EpL1pC44p	Freq. of CD45-
	EpCAM+	CD44+L1CAM-	EpL1nC44p	Freq. of CD45-
		CD44-L1CAM-	EpL1nC44n	Freq. of CD45-
		CD133-L1CAM+	EpC133nL1p	Freq. of CD45-
		CD133+L1CAM+	EpC133pL1p	Freq. of CD45-
		CD133+L1CAM-	EpC133pL1n	Freq. of CD45-
		CD133-L1CAM-	EpC133nL1n	Freq. of CD45-
		CD44+	CD44pEp	Freq. of CD45-
CD45-	EpCAM-	EnC45n	Freq. of alive	
		CD44-CD133+	EnC133pC44n	Freq. of CD45-
		CD44+CD133+	EnC133pC44p	Freq. of CD45-
		CD44+CD133-	EnC133nC44p	Freq. of CD45-
		CD44-CD133-	EnC133nC44n	Freq. of CD45-
		CD44-L1CAM+	EnL1pC44n	Freq. of CD45-
	EpCAM-	CD44+L1CAM+	EnL1pC44p	Freq. of CD45-
		CD44+L1CAM-	EnL1nC44p	Freq. of CD45-
		CD44-L1CAM-	EnL1nC44n	Freq. of CD45-
		CD133-L1CAM+	EnC133nL1p	Freq. of CD45-
		CD133+L1CAM+	EnC133pL1p	Freq. of CD45-
		CD133+L1CAM-	EnC133pL1n	Freq. of CD45-
		CD133-L1CAM-	EnC133nL1n	Freq. of CD45-
		CD44+	CD44pEn	Freq. of CD45-

[*] only for ascites samples, in tissues: frequency of CD45-.
